# Meniscus surface texture is associated with degenerative changes in biological and biomechanical properties

**DOI:** 10.1038/s41598-022-16206-2

**Published:** 2022-07-13

**Authors:** Shunya Otani, Takashi Kanamoto, Shohei Oyama, Satoshi Yamakawa, Wen Shi, Ryo Miyazaki, Masaharu Aihara, Shiro Oka, Sanae Kuroda, Tsuyoshi Nakai, Keisuke Takenaka, Yuji Sato, Masahiro Tsukamoto, Akira Tsujii, Kosuke Ebina, Seiji Okada, Ken Nakata

**Affiliations:** 1 Department of Medicine for Sports and Performing Arts, Osaka University Graduate School of Medicine, 2-2 Yamadaoka, Suita, Osaka 565-0871 Japan; 2 Department of Musculoskeletal Regenerative Medicine, Osaka University Graduate School of Medicine, 2-2 Yamadaoka, Suita, Osaka 565-0871 Japan; 3 Department of Sports Medical Biomechanics, Osaka University Graduate School of Medicine, 2-2 Yamadaoka, Suita, Osaka 565-0871 Japan; 4 Department of Orthopedic Surgery, Aihara Hospital, 3-4-30 Makiochi, Minoh, Osaka 562-0004 Japan; 5 Department of Orthopedic Surgery, Itami City Hospital, 1-100 Koyaike, Itami, Hyogo 664-8540 Japan; 6 Department of Laser Process, Joining and Welding Research Institution, Osaka University, 11-1 Mihogaoka, Ibaraki, Osaka 567-0047 Japan; 7 Department of Orthopedic Surgery, Osaka University Graduate School of Medicine, 2-2 Yamadaoka, Suita, Osaka 565-0871 Japan

**Keywords:** Musculoskeletal system, Diagnosis, Cartilage

## Abstract

Meniscal degeneration is defined by semi-quantitative assessment of multiple histological findings and has been implicated in biomechanical dysfunction, yet little is known about its relationship with biological properties. This paper aimed to quantitatively evaluate degenerative findings in human meniscus to examine their relationship with gene expression and biomechanical properties, and to extract histological findings that reflect biological properties like gene expression and cytokine secretion. This study included lateral menisci of 29 patients who underwent total knee arthroplasty. The menisci were divided into six samples. For each sample, Pauli's histological evaluation and corresponding quantitative assessment (surface roughness, DNA content, collagen orientation, and GAG content) were performed, with surface roughness showing the highest correlation with the histological evaluation in a single correlation analysis (r = 0.66, *p* < 0.0001) and multivariate analysis (*p* < 0.0001). Furthermore, surface roughness was associated with gene expression related to meniscal degeneration and with tangent modulus which decreases with increasing degeneration (r = − 0.49, *p* = 0.0002). When meniscal tissue was classified by surface integrity, inflammatory cytokine secretion tended to be higher in severe degenerated menisci. These results suggest that the evaluation of meniscal surface texture could predict the degree of degeneration and inflammatory cytokine secretion.

## Introduction

Knee osteoarthritis (OA) is a common disease with a large number of patients worldwide^1^, and the involvement of various tissues in knee joint has been suggested^2–4^. The meniscus plays an important role in load distribution and shock absorption^5,6^, and biomechanical dysfunction may contribute to OA progression^7–9^. Meniscal dysfunction is caused by traumatic injuries^10^ and degeneration due to the accumulation of microdamage, inflammation, and aging^11,12^. Recently, much attention has been paid to tissue changes and related molecular mechanisms underlying meniscal injury and degeneration.

Histological evaluation is commonly used to assess the tissue quality, and Pauli's histological grading system is frequently used for evaluating degeneration of the meniscus^13^. This system was developed based on previous reports reviewing meniscal histological findings^14,15^ and has four evaluation criteria: surface integrity, cellularity, collagen organization, and safranin-O staining, allowing for systematic evaluation of changes in aging and OA. However, it is a subjective and semi-quantitative evaluation, and the relationship between each criterion and function remains unclear. Furthermore, there are no reports on the reliability of any of the four criteria. For the articular cartilage degeneration, Osteoarthritis Research Society International (OARSI) histopathological grading system, which aims to simplify and improve reproducibility, is now widely utilized^16^. For the meniscus, it would be desirable to establish a more objective and quantitative histological evaluation method. In the Pauli's histological grading system, surface integrity accounts for half of total score, and furthermore, in the macroscopic grading system, it is assessed by the presence of gross surface fibrillation, suggesting that it is valuable to assess meniscal degeneration by measuring surface texture.

In recent years, it has been reported that the meniscus may be biologically involved in OA progression^17,18^. Regarding biomechanical properties, several important findings have been obtained^19,20^. It was found that meniscal degeneration is associated with histological changes and biomechanical properties^21^. The importance of inflammatory factors in OA progression has long been recognized^11,22^. The biological properties of articular cartilage and synovium have been extensively investigated^3,22,23^. Yet, very little has been reported on the meniscus, and no reports have been found that examine the relationship between the biological properties such as cytokine production and histological changes caused by degeneration.

It was hypothesized that histological findings of meniscal tissue are associated with gene expression and cytokine secretion and that surface texture is a valuable indicator of meniscal degeneration. The purpose of this study was to quantitatively evaluate the criteria used for assessing histological degeneration of the human meniscus, and to correlate these quantitative values with histological score, gene expression, and biomechanical property. This would allow us to extract a criterion that enables simple histological evaluation of degeneration, and to investigate whether meniscal degeneration is involved in changes in biological properties such as gene expression and cytokine secretion associated with OA progression.

## Results

### Meniscus surface roughness reflects Pauli’s histological grading system

**Figure 1:**
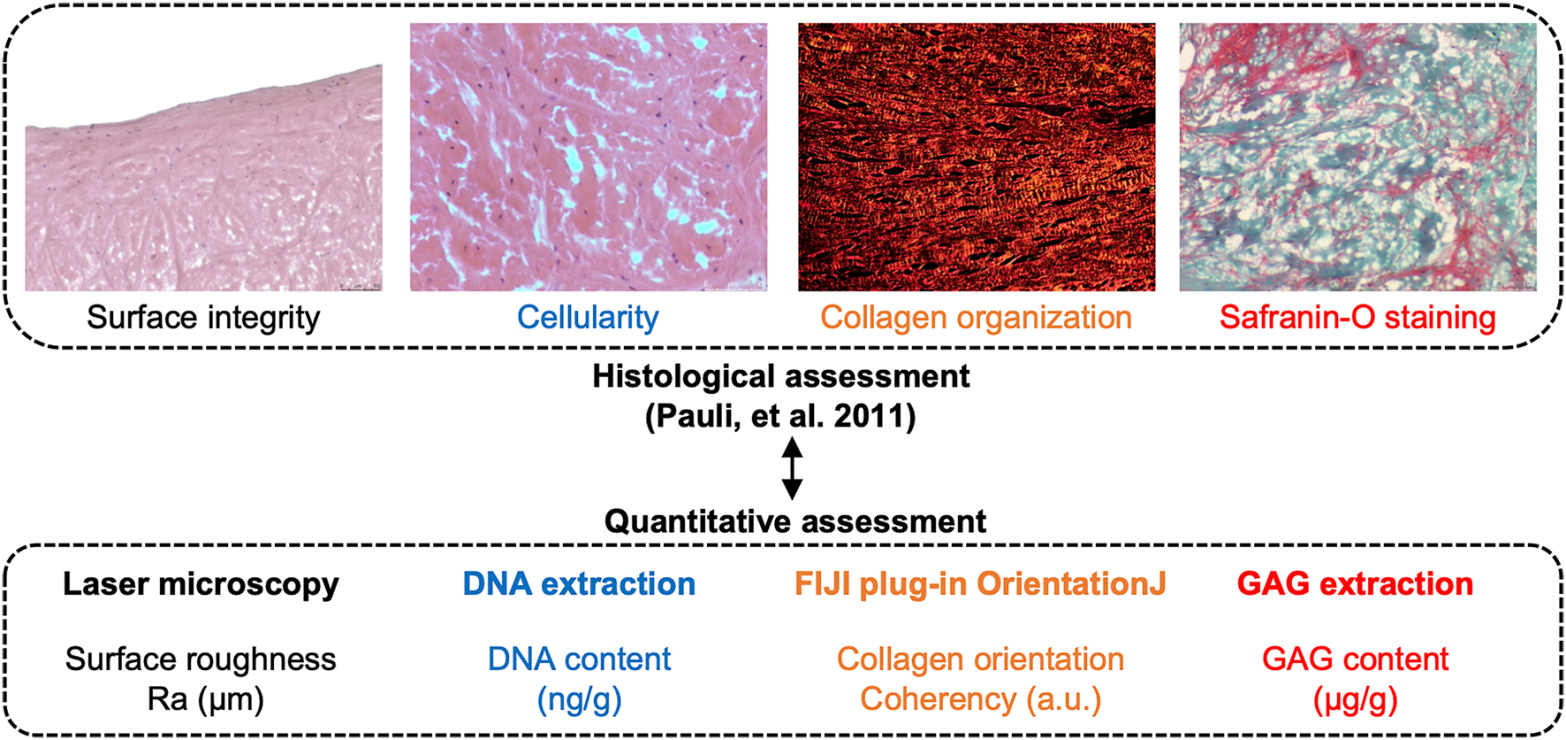
Comparison of Pauli’s histological grading system with surface roughness, DNA content, collagen orientation, and GAG content. Criteria used in Pauli’s histological grading system and the corresponding quantitative assessment. In quantitative assessment, the measurement methods, parameters, and units are described. Ra; arithmetical mean roughness.

**Figure 2:**
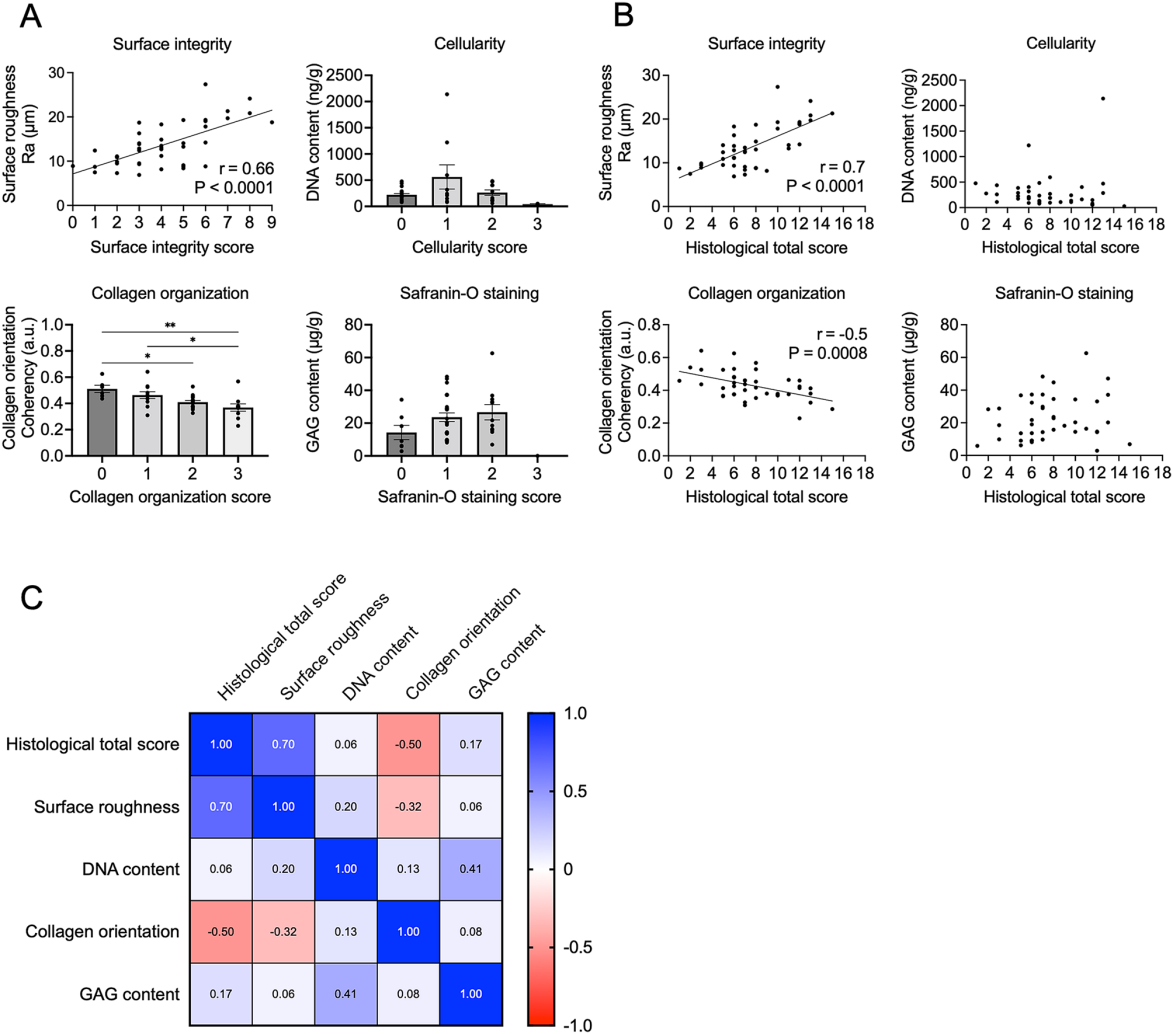
Relationship between Pauli’s histological evaluation and quantitative assessment of human meniscus. A total of 42 samples from seven menisci were included in each study. (**A**) Comparison between histological criteria and four corresponding quantitative values. *p < 0.05. Values are shown as mean ± SEM. (**B**) Correlation between the total histological score and four quantitative values. (**C**) Correlation matrix showing correlation coefficients among variables.

This study first compared Pauli’s histological evaluation criteria with corresponding quantitative assessment values (Fig. 1). The distribution of samples by histological evaluation is shown in Table S1. Inter-rater Intraclass correlation coefficient (ICC) for Pauli’s total histological score was 0.974. The ICCs of each criterion were 0.976, 0.947, and 0.948 for surface integrity, collagen organization, and safranin-O staining scores, respectively, while the ICC for cellularity score was 0.81, which was lower than that of the other criteria (Table 1). The strongest correlation was observed for the surface integrity score and surface roughness measured by laser microscopy (r = 0.66 , R^2^ = 0.44, *p* < 0.0001). As for collagen organization score, higher coherency was observed in scores of 0 (mean, 0.51; 95% CI, 0.44–0.58) and 1 (mean, 0.46; 95% CI, 0.41–0.52) than in score 3 (mean, 0.37; 95% CI, 0.31–0.43). No significant differences were observed in cellularity and safranin-O staining scores (Fig. 2A). In the correlation between each quantitative assessment and total histological score, surface roughness showed a strong positive correlation (r = 0.7, R^2^ = 0.49, *p* < 0.0001). As for collagen orientation, a moderate correlation was observed (r = -0.5, R^2^ = 0.25, *p* = 0.0008), while DNA and GAG content were not significantly correlated (Fig. 2B,C). Moreover, multiple regression analysis proved that surface roughness (*p* < 0.0001) and collagen orientation (*p* = 0.009) were related to histological total score (Table 2). These results indicate that the surface roughness obtained by quantitative surface texture measurement may best reflect meniscal histological degeneration.

**Table 1:** Inter-rater intraclass correlation coefficient (ICC) in each histological score.

	Total score	Surface integrity	Cellularity	Collagen organization	Safranin-O staining
ICC	0.974	0.976	0.81	0.947	0.948

**Table 2:** Multiple linear regression analysis of quantitative assessment values and total histological score.

Quantitative assessment	Estimate (95% CI)	t value	*P* value
Surface roughness	0.4 (0.25 to 0.55)	5.42	< 0.0001
DNA content	− 0.0009 (− 0.003 to 0.001)	0.83	0.41
Collagen orientation	− 11.88 (− 20.62 to − 3.13)	2.75	0.009
GAG content	0.047 (− 0.008 to 0.1)	1.73	0.09

### Surface roughness is correlated with gene expression and biomechanical property

**Figure 3:**
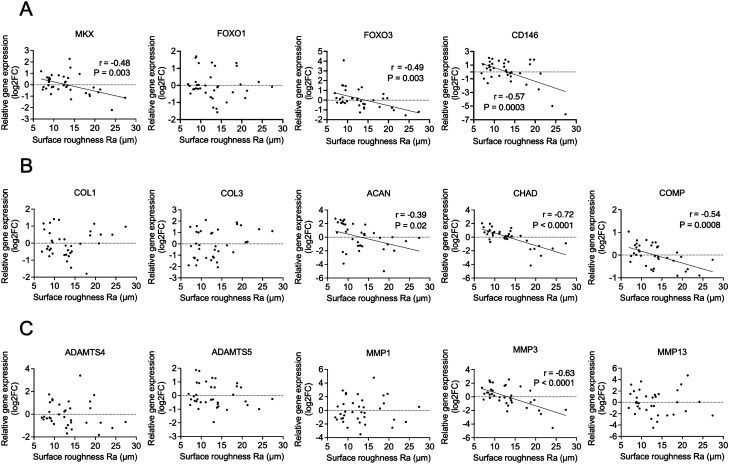
Correlation of surface roughness with gene expression in the meniscus. A total of 36 samples from six menisci were included in each study. (**A**) Correlation with meniscus degeneration-related gene expression. (**B**) Correlation with meniscus matrix gene expression. (**C**) Correlation with matrix degradative enzyme gene expression.

**Figure 4:**
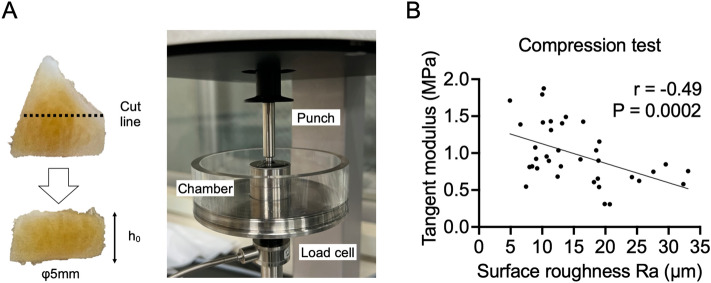
Correlation of surface roughness with biomechanical property in the meniscus. A total of 36 samples from six menisci were included in each study. (**A**) Sample preparation and apparatus used for compression test. (**B**) Correlation between surface roughness and tangent modulus.

According to multivariate analyses, surface roughness and collagen orientation were correlated with histological meniscal degeneration. Therefore, the relationship between these quantitative findings and gene expression was examined. In this study, Mohawk (*MKX*), Forkhead Box O1 (*FOXO1*), *FOXO3*, and *CD146*, which are genes that have been reported to be altered by meniscal degeneration based on previous reports^24–26^, were included in the analysis, in addition to the meniscus matrix and matrix degradative enzyme. These meniscus degeneration-related gene expression was correlated with histological total score (Fig. S2). *MKX* (r = − 0.48, R^2^ = 0.23, *p* = 0.003), *FOXO3* (r = − 0.49, R^2^ = 0.24, *p* = 0.003), and *CD146* (r = − 0.57, R^2^ = 0.32, *p* = 0.0003) showed a significant negative correlation with surface roughness. However, *FOXO1* expression showed no correlation (Fig. 3A). Regarding to meniscus matrix and degradative enzyme gene expression, *ACAN* (r = − 0.39, R^2^ = 0.15, *p* = 0.02)*, CHAD* (r = − 0.72, R^2^ = 0.52, *p* < 0.0001)*, COMP* (r = − 0.54, R^2^ = 0.29, *p* = 0.0008), and *MMP3* (r = − 0.63, R^2^ = 0.4, *p* < 0.0001) showed a significant negative correlation with surface roughness (Fig. 3B,C). In contrast, none of the meniscus degeneration-related genes showed a significant correlation with collagen orientation (Fig. S3A). A significant correlation was observed for *COMP* (r = 0.49, R^2^ = 0.24, *p* = 0.003) and *MMP3* (r = 0.45, R^2^ = 0.20, *p* = 0.006) (Fig. S3B and S3C). For surface roughness, the correlation with biomechanical properties was also examined (Fig. 4A). Tangent modulus measured by the compression test demonstrated negative correlation with surface roughness (r = − 0.49, R^2^ = 0.24, *p* = 0.0002) (Fig. 4B). These results demonstrate that surface roughness is correlated with changes in biological and biomechanical properties associated with meniscal degeneration.

### Relationship between surface integrity, a histological surface texture measurement, and OA-related cytokine production

**Figure 5:**
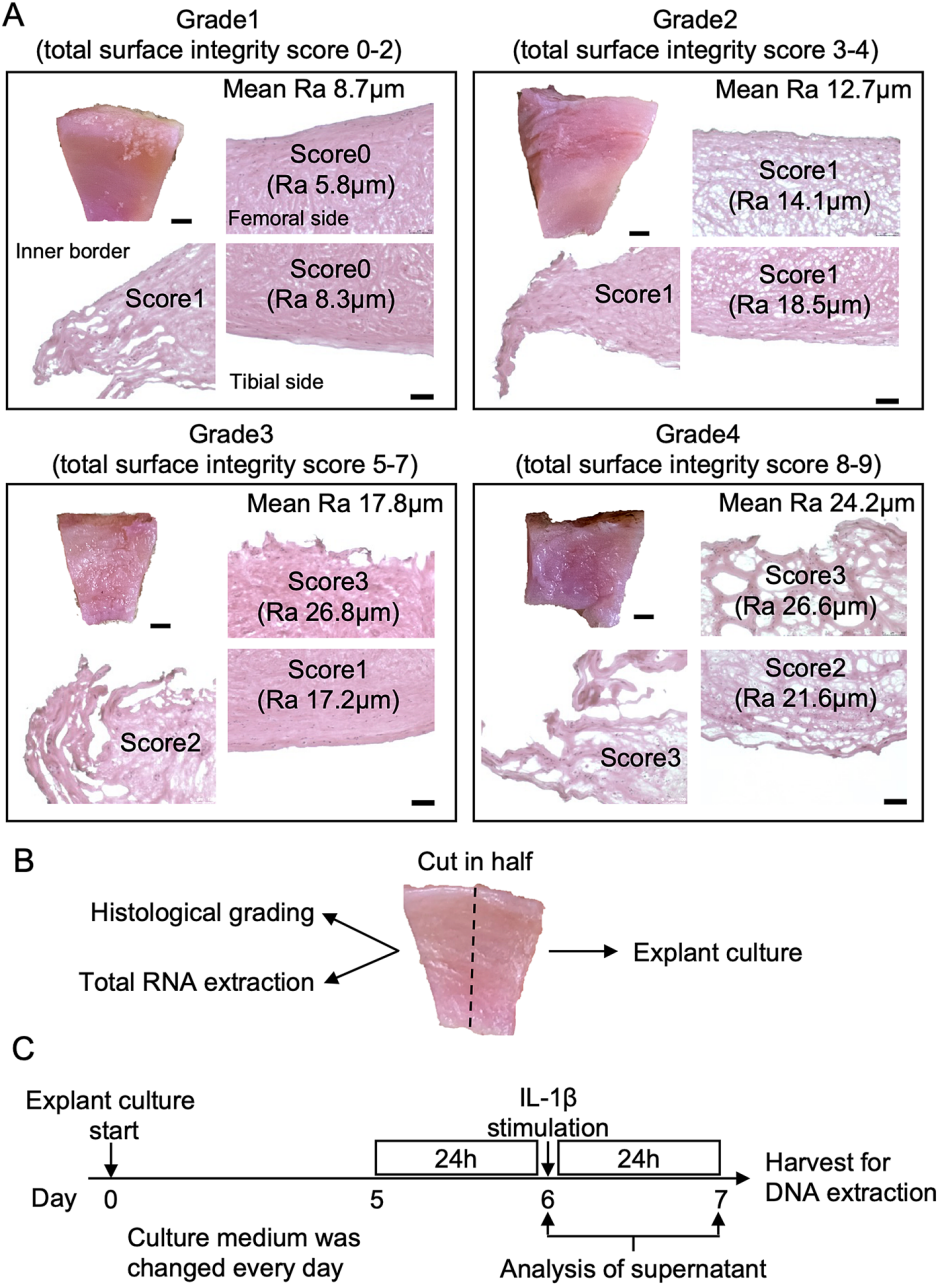
Investigation of the usefulness of meniscal tissue classification based on histological finding, surface integrity. (**A**) Representative images of different surface integrity grades in the meniscus, visible both in macroscopic and histological findings. Scale bars: 2 mm (macro), 100 μm (H&E). (**B**) Sample preparation. (**C**) Schedule of explant culture.

Based on these results, the degree of meniscal degeneration was classified histologically based on surface integrity alone (Fig. 5A, Table S2), and the relationship to gene expression and protein production of OA-related cytokines was investigated (Figs. 5B,C). The relationship between these cytokine secretion and histological total score was shown in Fig. S4.

**Figure 6:**
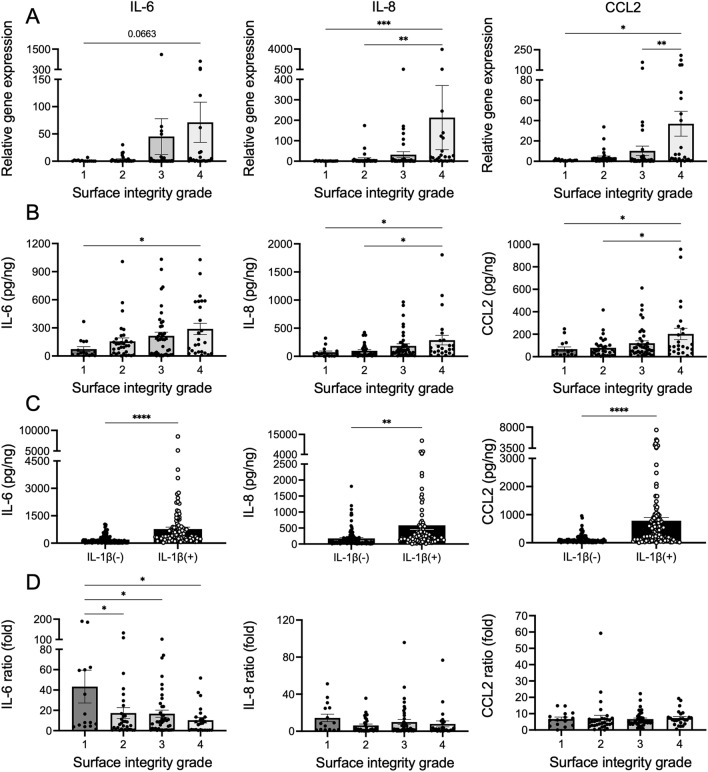
Relationship between surface integrity grade and OA-related cytokines. A total of 114 samples from 19 human menisci were included in each study. (**A**) Level of gene expression. (**B**) Protein concentration before IL-1β stimulation. (**C**) Comparison of protein concentration before and after IL-1β stimulation. (**D**) Change in protein concentration. *p < 0.05; **p < 0.01; ***p < 0.001; ****p < 0.0001. Values are shown as mean ± SEM.

Regarding gene expression, IL-8 and CCL-2 were highly expressed in grade 4 menisci compared to that in grade 1 menisci. (*p* = 0.0003 and 0.02, respectively) (Fig. 6A). Similarly, protein content in the supernatant before IL-1β stimulation was significantly higher in grade 4 menisci than in grade 1 menisci (IL-6, *p* = 0.04; IL-8, *p* = 0.05; CCL2, *p* = 0.04) (Fig. 6B). Furthermore, the response to IL-1β stimulation, which plays an important role in OA progression, was also examined. All these proteins were significantly increased by IL-1β stimulation (Fig. 6C). A comparison of protein production before and after IL-1β stimulation for each grade showed a significant difference in IL-6 levels depending on the degree of degeneration (grade 1, 43.3-fold; grade 2, 17.3-fold; grade 3, 16.7-fold; grade 4, 10.3-fold) (Fig. 6D). For IL-8 and CCL2, no significant differences among grades were observed.

## Discussion

The most important finding of this study is that quantitative assessment of four criteria utilized for meniscal degenerative evaluation showed that surface roughness measured by laser microscopy was highly correlated with total histological score and related to gene expression associated with meniscal degeneration. Furthermore, tangent modulus also correlated with surface roughness of the meniscus. Based on these results, surface integrity, a simple histological assessment of surface texture, was used to evaluate meniscal degeneration, demonstrating that surface integrity reflects meniscal biological properties such as OA-related cytokine secretion.

As a result of investigating the inter-rater ICC of Pauli's histological criteria and correlating them with the corresponding quantitative assessment, surface integrity score showed a high ICC, and a high correlation with surface roughness, which is a quantitative surface texture assessment. arithmetical mean roughness (Ra) values were also highly correlated with total histological score. Changes in the meniscus surface with aging have been observed in previous reports^27^, but there have been no detailed studies on changes associated with degeneration. Laser microscopy is commonly used to evaluate the surface texture of materials^28,29^. As for the tissue evaluation, although there is a report on its use in the cornea^30^, this study is the first attempt to evaluate the meniscus.

*MKX, FOXO1, FOXO3,* and *CD146* have been reported to be marker genes for degenerative changes in the meniscus^24–26^. Transfection of *MKX* into human degenerative meniscus explants has been shown to improve histological scores and reduce catabolic, hypertrophic, and calcification gene expression^24^. Regarding *FOXO1* and *FOXO3,* menisci obtained from a mouse with loss-of-function showed a worse histological score^25^. Moreover, *CD146* is downregulated in cells isolated from degenerative menisci^26^. As for matrix of the meniscus, this study showed a negative correlation of surface roughness with *ACAN, CHAD,* and *COMP* gene expression. These proteoglycans were reported to decrease in human OA menisci^31^. With respect to matrix degradative enzyme, *MMP3* gene expression level was negatively correlated with surface roughness. For the articular cartilage, the highest expression levels of *MMP3* were reported in normal and early degenerated human cartilage, while a significant decrease in expression was observed in late OA specimens^32,33^. In the current study, Ra values were negatively correlated with the above gene expression, and also negatively correlated with meniscal tangent modulus, suggesting that meniscal surface roughness is associated with, and could be an indicator of, tissue degeneration. Based on these results, this study performed a more useful and simple histological evaluation of meniscal degeneration by using surface integrity, which is one of Pauli's histological evaluation criteria.

Several reports have been published on synovium^18^, articular cartilage^34,35^, and subchondral bone^36^ as sources of cytokines involved in OA pathogenesis. Several studies have examined catabolic gene expression levels in menisci extracted at the time of meniscectomy^37^ and found that menisci from OA knees have the same cytokine production capacity as synovium^38^. The findings of this study suggest that meniscus, like other intra-articular tissues, may be a source of cytokine secretion. Furthermore, the relationship between the degree of meniscal degeneration and cytokine secretion showed that a severely degenerative meniscus secreted more cytokines. The relationship between histological degeneration and cytokine production has not been reported previously, and the results of this study provide novel insights regarding this connection. These findings suggest that meniscal degeneration may be biologically involved in OA progression. Regarding the response of meniscal tissue to IL-1β stimulation, increased secretion of cytokines has been reported in an ex vivo canine model^17^, which is consistent with the results of human ex vivo model in this study. As for the relationship with the degree of degeneration, the responses differed among the proteins; therefore, further studies are needed. Regardless of the degree of degeneration, the meniscus secretes cytokines in response to IL-1β stimulation, suggesting the importance of chronic inflammation in knee joints with relatively mild degeneration.

This study had certain limitations. First, all samples were lateral menisci from patients with OA or osteonecrosis, and this study included no data from the medial meniscus or healthy normal meniscus. Second, for biomechanical properties, this study only investigated correlations with surface roughness and did not evaluate correlations with other criteria. Finally, it is unclear whether the cytokines secreted from the meniscus affect OA progression. Further verification using animal and co-culture models^39^ is necessary.

This study has two clinical implications. First, the results of this study have shown the possibility of predicting the biomechanical and biological properties of meniscal tissue by evaluating its surface texture. As with articular cartilage injuries^40,41^, this is a novel finding that evaluation of the surface texture of the meniscus by knee arthroscopy and MRI imaging findings can be an important criterion for pathological evaluation and treatment selection. In the future, further investigation is needed to determine whether arthroscopic observation of surface texture can be applied to evaluate meniscal degeneration. Second, this study found that meniscal degeneration may be a biological factor in the progression of OA. To date, treatment to maintain and improve the biomechanical function of meniscal tissue has been sought^19,42^. In recent years, interventions aimed at improving its biological properties are also performed^43^.

In summary, the surface roughness of meniscal tissue reflects the biological and biomechanical properties, and the surface integrity, a histological surface texture evaluation, reflects the OA-related cytokine secretion, which is increased with histologically severe meniscal degeneration. This novel insight will be helpful in considering the involvement of the meniscus in the pathogenesis of OA and in developing interventions to address it.

## Materials and methods

### Ethics statement

This study was conducted using human meniscal tissues and was approved by the Osaka University Institutional Ethical Committee (approval ID: 16,085–4). Written informed consent was obtained from all patients, and all the methods were performed in accordance with the relevant guidelines and regulations.

### Sample preparation

This study analyzed 32 lateral menisci from 29 patients (mean age, 75.1 years; range, 59–90 years; 5 men and 24 women) with knee OA or osteonecrosis who underwent total knee arthroplasty. The meniscus was processed within 48 h after surgery and divided into six samples (A, Am, Ma, Mp, Pm, and P) for histological evaluation (Fig. S5A), quantitative assessment, gene expression analysis, and protein secretion analysis. For the compression test, the samples were drilled using a disposable biopsy punch (diameter = 5 mm; Kai Medical, Solingen, Deutschland). For protein secretion analysis, each sample was cut in half vertically, where one half was cultured in Dulbecco’s modified Eagle’s medium/high glucose (Sigma-Aldrich, St. Louis, MO, USA) with 1% penicillin/streptomycin, and incubated at 37 °C, 90% humidity, and 5% CO_2_. The other half was used for histological and gene expression analyses. Except for protein secretion analysis, all samples were frozen at − 80 °C until use.

### Histological evaluation

Vertical and horizontal cryosections of 10 μm were prepared by cutting them in two different planes. Vertical sections were stained with hematoxylin and eosin (H&E) and safranin-O/fast green to evaluate surface integrity, cellularity, and glycosaminoglycan (GAG) staining, while horizontal sections were stained with safranin-O/fast green and picrosirius red to evaluate collagen organization. Images were obtained using a DMi8 microscope (Leica, Germany) or BX53/DP74 (Olympus, Tokyo, Japan). Pauli’s histological grading system was used to evaluate the degree of degeneration. The evaluation was performed by three investigators (S. O., R. M. and T. K.). ICC was used to evaluate inter-rater agreement.

### Surface roughness measurement

The thawed samples were subjected to surface roughness analysis using a shape-measurement laser microscope (VK-X200; Keyence, Osaka, Japan) with a 10 × objective lens. Ra at four areas (the outer and inner surfaces of the femoral and tibial sides) was measured based on the JIS B0601:2001 (ISO 4287:1997) surface texture parameters. Ra is the average absolute value of the sample length. For each sample, a surface section of 1420 μm × 1065 μm was scanned (Fig. S5B).

### DNA content measurement

Meniscal DNA was extracted using a PureLink Genomic DNA Purification Kit (Thermo Fisher Scientific, Waltham, MA, USA) according to the manufacturer’s instructions. Prior to enzymatic digestion, each sample was weighed to normalize DNA content. DNA concentration was measured using a Qubit 4.0 fluorometer (Thermo Fisher Scientific).

### Collagen orientation assessment

Slides stained with picrosirius red were imaged using a BX53/DP74 microscope (Olympus) equipped with a polarization filter. Images were obtained from one location, each in the inner and outer areas. The FIJI plug-in OrientationJ was used to evaluate collagen orientation. The images were converted to 32-bit grayscale, and coherency was determined for a random set of 100 regions of interest (ROIs, 200 pixels) per image (Fig. S5C). Coherency = 1 indicates that the collagen fibers have one dominant orientation.

### GAG content measurement

Meniscal samples were digested with papain (Sigma-Aldrich; 125 μg/ml) at 65 °C overnight, and meniscal GAG was extracted using the Blyscan Glycosaminoglycan Assay Kit (Biocolor Ltd., UK) according to the manufacturer’s instructions. Prior to enzymatic digestion, each sample was weighed to normalize the GAG content. The absorbance at 656 nm was measured using a microplate reader (MultiSkan GO; Thermo Fisher Scientific).

### RNA extraction and quantitative real-time polymerase chain reaction (qPCR)

Total RNA was isolated from meniscal tissue using TRIzol (Invitrogen) and a PureLink RNA Purification kit (Thermo Fisher Scientific) and reverse-transcribed using a High-Capacity RNA-to-cDNA kit (Thermo Fisher Scientific) to synthesize complementary DNA. qRT-PCR was performed using Power SYBR Green Master Mix and QuantiStudio 7 Pro Real-Time PCR System (Thermo Fisher Scientific) according to the manufacturer’s instructions. Specific primers were used for target genes (Table S3). The ΔΔCT method was used to evaluate gene expression, which was normalized to GAPDH.

### Compression test

Following a previous study^17^, cylindrical samples were obtained from the center of each sample using a biopsy punch (φ5 mm) and cut parallel to tibial surface. Subsequently, the height (h_0_) was measured using a digital caliper (CD-10AX; Mitutoyo, Japan, 0.01 mm resolution, ± 0.02 mm maximum permissible error). Biomechanical property analysis under confined compression conditions was conducted using a test instrument (ElectroForce 5500; TA Instruments, New Castle, DE, USA) equipped with a stainless steel punch. The samples were placed in a cylindrical metallic chamber filled with PBS. Following the application of a preload (0.1 N), the samples were loaded to a 20% strain level. The loading rate was 3% h_0_/min. Stress–strain curve was described, and the tangent modulus was calculated from the liner region.

### IL-1β stimulation

Human recombinant interleukin (IL) -1β (5 ng/ml; R&D Systems, Minneapolis, MN, USA) was added 6 days after the start of explant culture. The supernatant was collected 24 h before and after IL-1β stimulation and was used for protein concentration measurements. Twenty-four hours after stimulation, the samples were harvested for DNA content measurement to normalize the protein concentration.

### Quantitative protein analysis using homogeneous time-resolved fluorescence (HTRF)

An enzyme immunoassay was performed to measure the concentration of IL-6, IL-8, and C–C motif chemokine ligand 2 (CCL2) using HTRF human assay kits (Cisbio). The supernatant was evaluated 24 h before and after IL-1β stimulation were evaluated, and the ratio of protein concentration before and after IL-1β stimulation was calculated. Protein concentration was normalized to total DNA content.

### Statistical analysis

Pearson’s correlation coefficient and an analysis of variance (ANOVA) with Tukey–Kramer post-hoc test were performed to analyze the relationship between histological score and quantitative values, correlation of quantitative values of surface roughness with gene expression and biomechanical properties, and protein concentration. Associations between histological total score and quantitative assessment values were evaluated with use of multiple linear regression analysis. Kruskal–Wallis test and Steel–Dwass post-hoc test were performed for multiple comparisons of gene expression. The change by IL-1β stimulation was analyzed using a paired t-test. Data are presented as the mean ± standard error of the mean (SEM). All statistical analyses were performed using GraphPad Prism 9 (San Diego, CA, USA) and statistical significance was defined as *p* < 0.05.

## Supplementary Information

Supplementary Information.

## Data Availability

The datasets used and/or analysed during the current study available from the corresponding author on reasonable request.
