# Supplementary material for: Meniscus surface texture is associated with degenerative changes in biological and biomechanical properties
Source: Sci Rep. 2022 Jul 13;12:11977. doi: 10.1038/s41598-022-16206-2 (PMC9279509; doi:10.1038/s41598-022-16206-2)
Supplement: Supplementary file 1 — Supplementary Information. [file 41598_2022_16206_MOESM1_ESM.pdf]

A

Pauli's total histological score	0	1	2	3	4	5	6	7	8	9	10	11	12	13	14	15	16	17	18
**Sample numbers**	0	1	1	3	0	4	8	6	5	1	3	2	4	3	0	1	0	0	0

B

Surface integrity score	0	1	2	3	4	5	6	7	8	9
**Sample numbers**	1	3	5	9	7	6	6	2	2	1

C

Cellularity score	0	1	2	3
**Sample numbers**	21	9	10	2

D

Collagen organization score	0	1	2	3
**Sample numbers**	6	11	15	10

E

Safranin-O staining score	0	1	2	3
**Sample numbers**	7	23	12	0

Supplementary Table S1. Sample distribution, related to Figures 2.

(A) Pauli's total histological score. (B) Surface integrity score. (C) Cellularity score.

(D) Collagen organization score. (E) Safranin-O staining score.

Surface integrity grade	Grade1	Grade2	Grade3	Grade4
**Region**				
**A**	0	2	6	11
**Am**	3	7	7	2
**Ma**	3	5	8	3
**Mp**	2	5	9	3
**Pm**	6	5	7	1
**P**	1	8	5	5
**Total**	15	32	42	25

Supplementary Table S2. Sample distribution, related to Figures 5 and 6.

Gene	Forward Primer	Reverse Primer
MKX	CTAGTCTCGAGGCGGGAGAG	TGTTCATGGTGTCGGTTCGT
FOXO1	AGGGTTAGTGAGCAGGTTACAC	CTGCACACATTGGGCAAACA
FOXO3	CATGAGAAGTTCCCCAGCGA	GTGTCAGTTTGAGGGTCTGCT
CD146	GGGGACTTTCAGCCTTGTGA	GACCAAGCTGCTTCGCAAAA
COL1	GTCGAGGGCCAAGACGAAG	CAGATCACGTCATCGCACAAC
COL3	GAGGACAGATTCTAGTGCTGAG	ATAGGTAGTCTCACAGCCTTGC
ACAN	TGAGGAGGGCTGGAACAAGTACC	GGAGGTGGTAATTGCAGGGAACA
CHAD	AAACAGGTTCTGACCCAGCC	GGAGCTGGGTTGGTAGTGGA
COMP	AGGGAGATCGTGCAGACAA	AGCTGGAGCTGTCCTGGTAG
ADAMTS4	GTGCCATTGTGGAGGATGAT	CTTGGAGTTGTCATGGAGCA
ADAMTS5	CTTGACTGTGGCTCACGAAA	TTTGGACCAGGGCTTAGATG
MMP1	ACCCTGGATAGGCAAGGGAT	TCTGTAAGGGTGGCGTTGTC
MMP3	TCCTACTGTTGCTGTGCGTG	AGGTTCATGCTGGTGTCCTC
MMP13	CTTCCCAACCGTATTGATGC	ACTTCTTTTGGAAGACCCAGTTC
IL-6	GTGTGAAAGCAGCAAAGAGG	CCTCAAACTCCAAAAGACCAG
IL-8	TGGCAGCCTTCCTGATTTC	GGGTGGAAAGGTTTGGAGTATG
CCL2	AGCAGCAAGTGTCCCAAAGA	TTTGCTTGTCCAGGTGGTCC
GAPDH	TCTCTGCTCCTCCTGTTCGAC	GTTGACTCCGACCTTCACCTTC

Supplementary Table S3. Primer sequences for qRT-PCR, related to Figures 3 and 6.

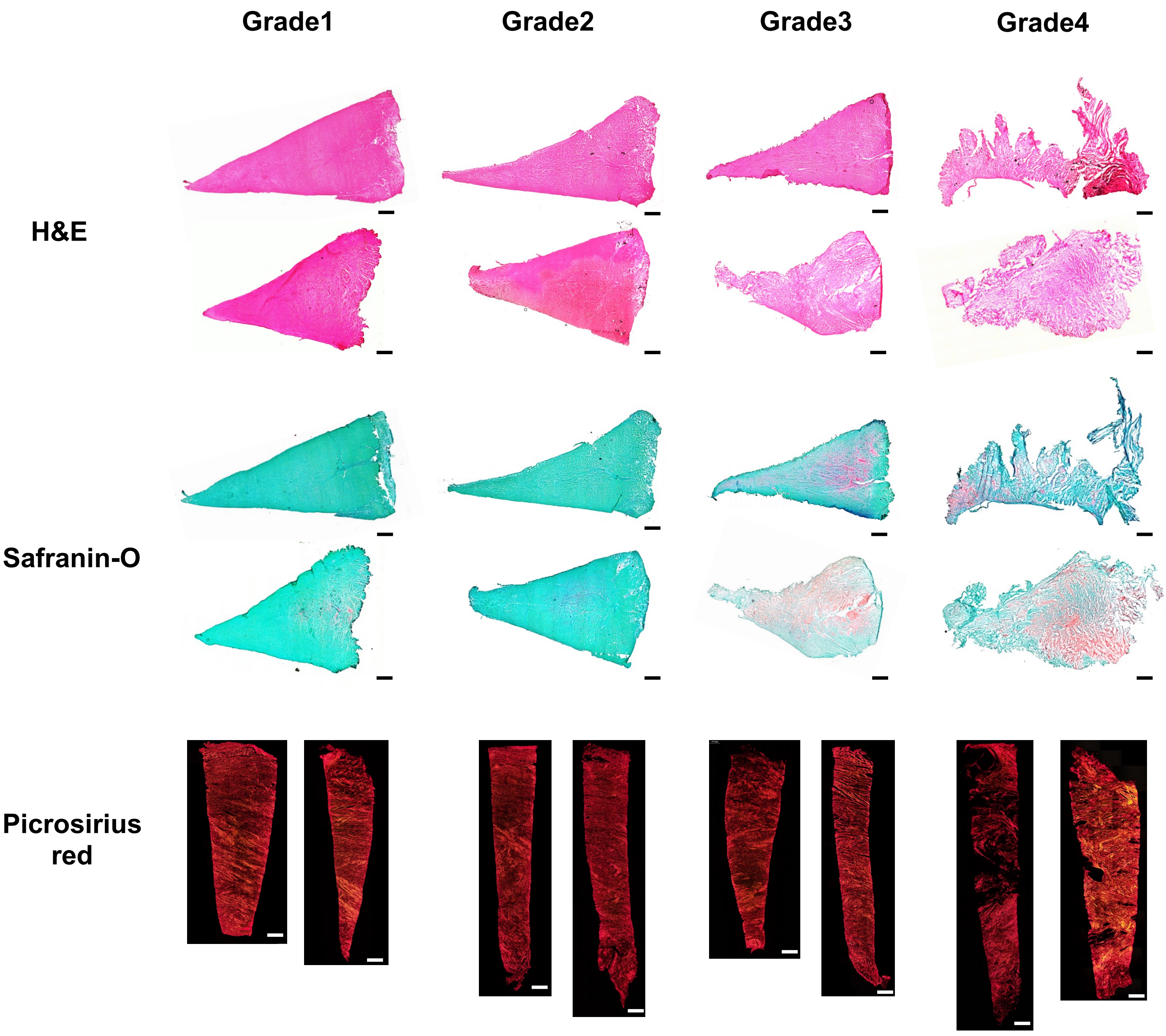

Supplementary Figure S1. Representative images of H&E, safranin-O, picrosirius red staining used for Pauli's histological evaluation. Scale bars: 1 mm

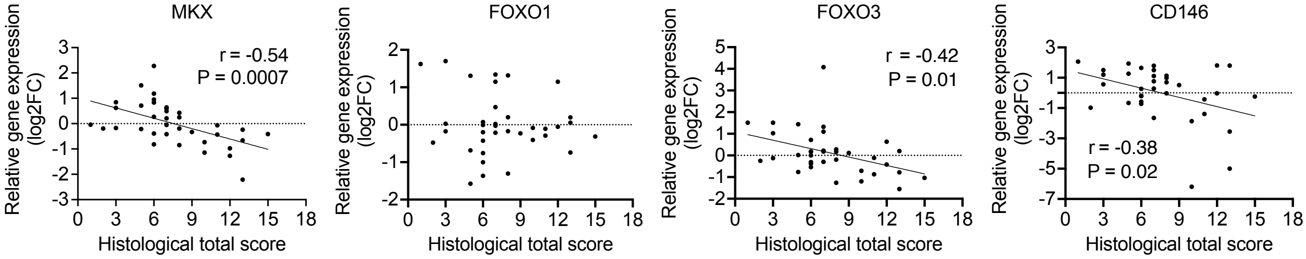

Supplementary Figure S2. Correlation of histological total score with meniscus degeneration-related gene expression.

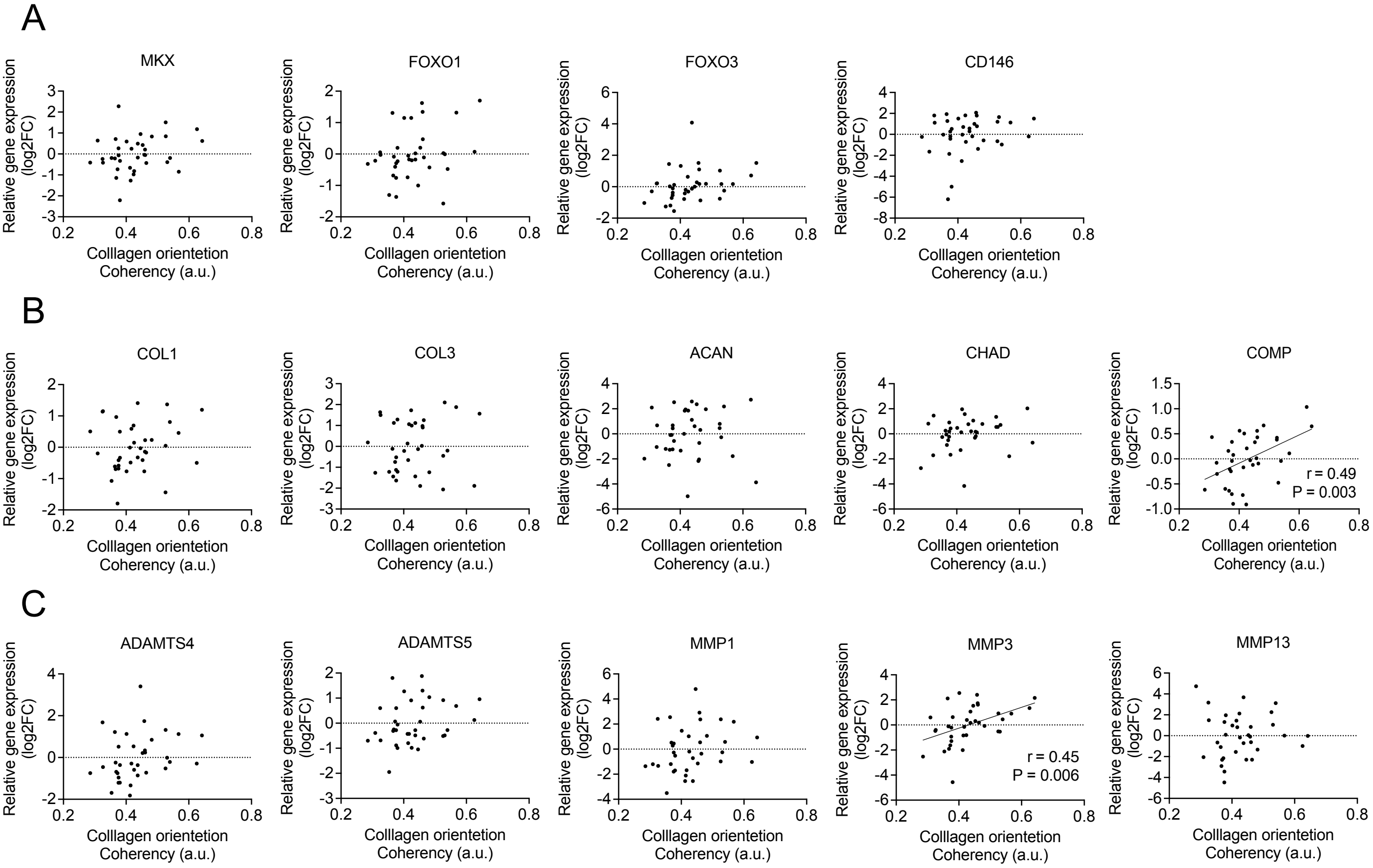

Supplementary Figure S3. Correlation of collagen orientation with gene expression.

(A) Correlation analysis with meniscus degeneration-related marker. (B) Correlation analysis with matrix of meniscus. (B) Correlation analysis with matrix degradative enzyme.

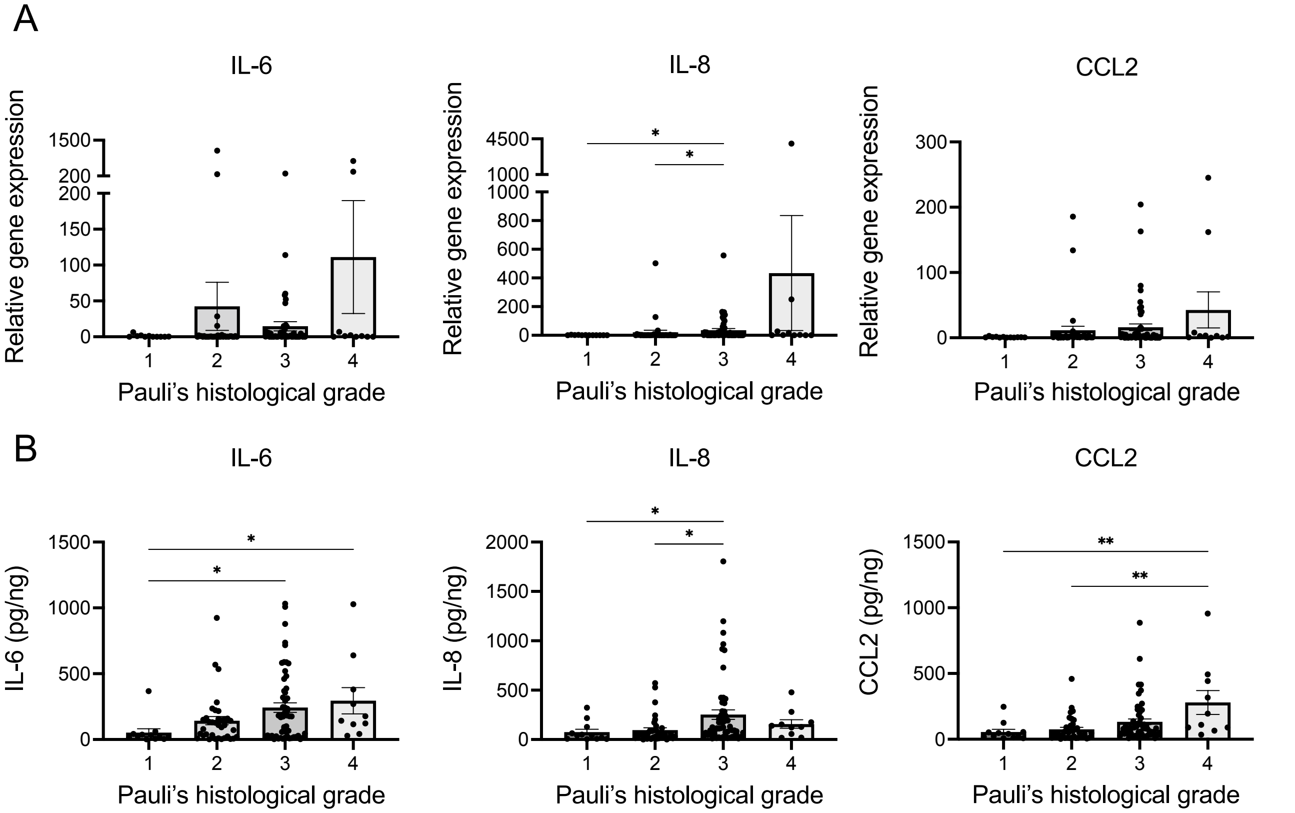

Supplementary Figure S4. Relationship between Pauli's histological grade and OA-related cytokines.

(A) Level of gene expression. (B) Protein concentration before IL-1β stimulation. *$p < 0.05$; **$p < 0.01$. Values are shown as mean ± SEM.

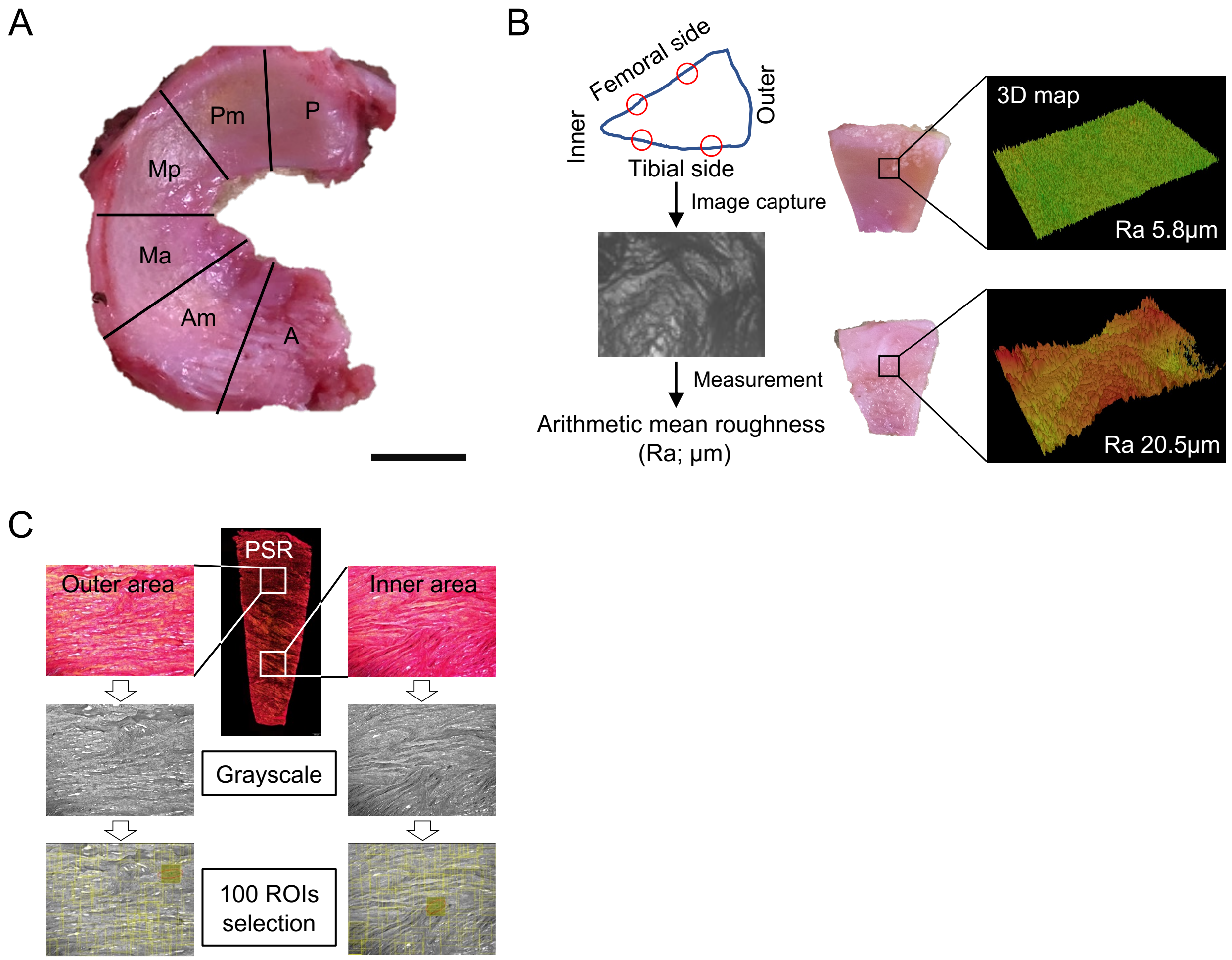

Supplementary Figure S5. Sample preparation and methods for quantitative assessment.

(A) Meniscus divided into six samples for further study. Scale bar: 10 mm (B) Schematic representation of the area for evaluating surface roughness at four locations (the outer and inner surfaces of the femoral and tibial sides). The 3D map shows representative data. (C) Evaluation protocol for collagen orientation analysis. PSR; Picrosirius Red staining, ROI; region of interest.
